# A systematic review of comorbidities and outcomes of adult patients with pleural infection

**DOI:** 10.1183/13993003.00541-2019

**Published:** 2019-10-01

**Authors:** Tamsin N. Cargill, Maged Hassan, John P. Corcoran, Elinor Harriss, Rachelle Asciak, Rachel M. Mercer, David J. McCracken, Eihab O. Bedawi, Najib M. Rahman

**Affiliations:** 1 Oxford Centre for Respiratory Medicine, Oxford University Hospitals NHS Trust, Oxford, UK; 2 Interventional Pulmonology Service, Respiratory Medicine Dept, University Hospitals Plymouth, Plymouth, UK; 3 Bodleian Health Care Libraries, University of Oxford, Oxford, UK; 4 Joint first authors

## Abstract

**Background:**

Pleural infection remains an important cause of mortality. This study aimed to investigate worldwide patterns of pre-existing comorbidities and clinical outcomes of patients with pleural infection.

**Methods:**

Studies reporting on adults with pleural infection between 2000 and 2017 were identified from a search of Embase and MEDLINE. Articles reporting exclusively on tuberculous, fungal or post-pneumonectomy infection were excluded. Two reviewers assessed 20 980 records for eligibility.

**Results:**

211 studies met the inclusion criteria. 134 articles (227 898 patients, mean age 52.8 years) reported comorbidity and/or outcome data. The majority of studies were retrospective observational cohorts (n=104, 78%) and the most common region of reporting was East Asia (n=33, 24%) followed by North America (n=27, 20%). 85 articles (50 756 patients) reported comorbidity. The median (interquartile range (IQR)) percentage prevalence of any comorbidity was 72% (58–83%), with respiratory illness (20%, 16–32%) and cardiac illness (19%, 15–27%) most commonly reported. 125 papers (192 298 patients) reported outcome data. The median (IQR) length of stay was 19 days (13–27 days) and median in-hospital or 30-day mortality was 4% (IQR 1–11%). In regions with high-income economies (n=100, 74%) patients were older (mean 56.5 *versus* 42.5 years, p<0.0001), but there were no significant differences in prevalence of pre-existing comorbidity nor in length of hospital stay or mortality.

**Conclusion:**

Patients with pleural infection have high levels of comorbidity and long hospital stays. Most reported data are from high-income economy settings. Data from lower-income regions is needed to better understand regional trends and enable optimal resource provision going forward.

## Abstract 2

**In pleural infection, patients from higher-income countries tend to be older with more comorbidities and are more likely to be referred for fibrinolytic treatment in comparison to patients from lower-income countries** http://bit.ly/2K2M5HL

## Introduction

Infection of the pleural space causes serious morbidity and is often life threatening [1]. Despite advances in management, 30-day mortality remains high, reported at between 9% and 10.5% in a recent Danish cohort [2]. This is especially true among older patients, in whom 30-day mortality has been reported at 20.2% in patients aged >80 years [2–4].

Pleural infection is common, with >30 000 diagnoses in the years 2000–2011 in the largest and most recent population-based cohort in Taiwan [5]. In recent years, incidence rates have been trending upwards [2, 3, 6], and coupled with advancing therapeutic techniques, the management of pleural infection represents a growing resource strain, with reported median length of hospital stay in a Canadian study averaging nearly 22 days [6]. The use of intrapleural fibrinolytics [7] as well as the improved safety profile for endoscopic thoracic surgery increased the average cost of hospitalisation in a Taiwan-based study to reach USD 4400 per admission in 2008, an increase of >60% over the preceding 12 years of the study [4].

The underlying drivers of the rise of pleural infection cases are not fully established. Possible mechanisms include the rise of multimorbidity in ageing populations, as well as immunosuppressive states such as HIV, predisposing individuals to the condition. This is supported by data from large population-based cohorts demonstrating that incidence is skewed towards older persons and is rising more quickly in this group [2, 6]. Furthermore, rates of comorbidity in pleural infection have been reported as being as high as 74% [2, 5], and patients with increased pre-existing comorbidity have higher mortality rates (20.6% if Charlton comorbidity score (CCS) >2 points, 6% if CCS 0 points) [2].

This tripartite trend of increasing incidence of pleural infection, accelerated cases among older persons and higher mortality among older and comorbid persons are consistently reported in large population-based cohorts from Canada, Taiwan, Denmark and USA [2–6]. However, these data do not necessarily represent worldwide patterns and to date no study has comprehensively reviewed the published data available. To address this, we performed a systematic review of the literature reporting the clinical characteristics and outcomes of patients with pleural infection, with a comparison between high-income and lower-income economies, of which reports from the latter are sparse. More research in low-economic settings will be essential going forward to understand regional trends and inform local resource provision.

## Methods

This review was performed according to Preferred Reporting Items for Systematic Reviews and Meta-Analyses (PRISMA) guidelines and the protocol registered on the PROSPERO international prospective register of systematic reviews (CRD42017076418) [8].

### Search strategy

Ovid MEDLINE and Embase were searched between 2000 and 2017 using the keywords “empyema”, “pleural infection” and “pleuritis”. The full search strategy is reported in detail elsewhere [8, 9].

### Data extraction

All records were screened independently by two authors (TC and MH).

The following inclusion criteria were used. 1) Population: adults (age >18 years) with bacterial pleural infection/empyema acquired in any setting (community, secondary or tertiary hospital care); 2) intervention: any intervention including conservative management with antibiotics and chest tube, intrapleural medication or any form of surgical procedure; 3) comparator: no comparator assessed; and 4) outcomes: in-hospital mortality, length of stay, escalation to surgical intervention in mixed cohort studies and any recorded comorbidity on admission.

Randomised and non-randomised controlled trials as well as observational or cross-sectional studies were included. Records with <20 participants were excluded due to the case selective nature of these reports.

Reports where over half of participants were aged <18 years or with tuberculous, fungal or post-pneumonectomy pleural infection were excluded, as the aetiology and outcome in these groups are not comparable to bacterial pleural infection in adults. Non-English language studies were included when suitable translation was available.

Data was extracted where available into a Microsoft Excel proforma. Countries of studies included in this review were classified by income as per the World Bank definition for the 2019 fiscal year [10] with gross national income (GNI) per capita in the year 2017 [11] as follows: low-income economies (GNI USD 995 per capita or less); lower-middle-income economies (GNI USD 996–3895 per capita); upper-middle-income economies (GNI USD 3896–12 055 per capita); high-income economies (GNI USD 12 055 or more per capita).

Collectively in this article low, lower-middle and upper-middle economies are referred to as lower-income economies.

### Subgroup analysis

Comorbidity data are reported as number and percentage of total participants in each study. Studies that specifically recruited patients with empyema and a specific disease exclusively (*e.g.* HIV) were excluded from the analysis of that particular comorbidity. Data were presented as prevalence of comorbidity both by affected organ/system and by specific disease.

### Outcome analysis

Data on 30-day/in-hospital mortality, length of hospital stay, need for surgical intervention and intrapleural fibrinolytic therapy were collected. Studies where an entire cohort was comprised of a single intervention were excluded from analysis of that particular outcome.

Statistical analysis was performed in Prism (version 8.0; GraphPad, San Diego, CA, USA). Median values were transformed where possible to means for the age variable using the formula: mean=((lower limit+(2×median)+upper limit))/4. A t-test for parametric data or Mann–Whitney U-test for non-parametric data was performed for statistical comparison between two groups.

## Results

### Cohort characteristics

**FIGURE 1:**
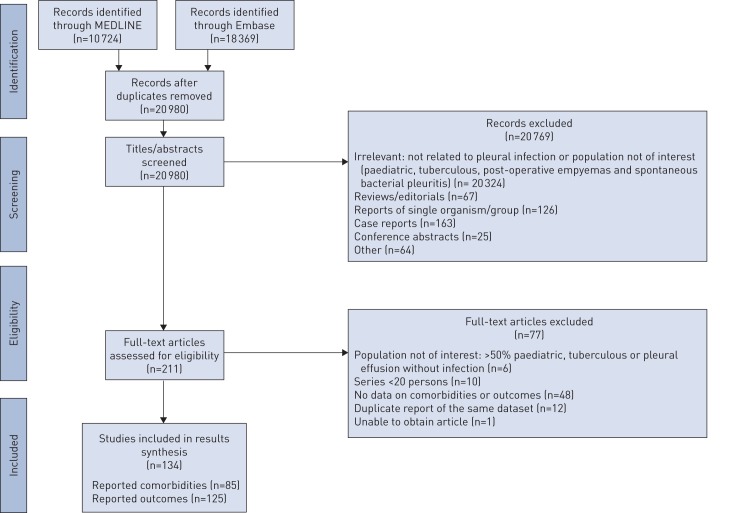
Preferred Reporting Items for Systematic Reviews and Meta-Analyses (PRISMA) flowchart showing the identification, screening, eligibility and inclusion process.

Of the 20 980 publications returned from the initial search, 211 studies met the inclusion criteria. 134 articles (totalling 227 898 patients) [2, 4–7, 12–140] reported comorbidity and/or outcome data (characteristics summarised in supplementary table S1). The remaining papers were excluded due to lack of relevant data (n=48), duplicate datasets (n=12), special populations predefined for exclusion in the protocol (n=6), case-series of <20 participants (n=10) or the original article was unobtainable (n=1), as shown in figure 1.

The majority of studies (n=104, 78%) were retrospective observational cohorts, while 17 (13%) studies were prospective observational studies, 11 (8%) were randomised controlled trials and two (2%) were diagnostic accuracy studies. The published data were skewed towards reports from high-income economies (n=100, 74%; totalling 224 476 patients) with over a quarter from low, low-middle and upper-middle economies combined (n=34, 26%; totalling 3422 patients, 1.5%). The most common region of reporting was East Asia (n=33, 24%) followed by North America (n=27, 20%).

The mean age of individuals in all studies was 52.8 years (95% CI 51.0–54.7 years). Patients in cohorts from high-income economies were significantly older than patients from lower-income economies (56.5 years *versus* 42.5 years, p<0.0001).

### Patients with pleural infection have a high prevalence of pre-existing comorbidity

85 articles reported comorbidity data (totalling 50 756 patients, summarised in supplementary table S2). The majority of published data were from countries with high-income economies (n=68, 79%; totalling 48 703 patients, 96%). Most reports were single-centre/multicentre retrospective or prospective observational cohorts (n=64, 74% and n=10, 12%, respectively). 38 studies were cohorts including empyema treated exclusively by surgery or fibrinolysis (n=27, 31% and n=11, 13%, respectively).

**FIGURE 2:**
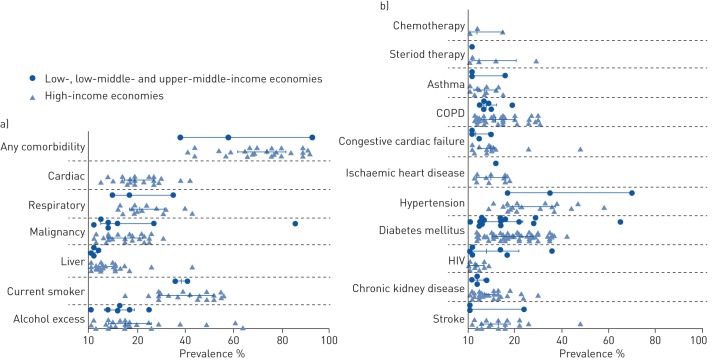
Pre-existing prevalence of comorbidity in studies of patients with pleural infection. Percentage prevalence of comorbidities in each study were extracted and data from high-income and lower-income economies were compared. a) Percentage prevalence of comorbidity, smoking, alcohol excess and disease by organ system affected; b) percentage prevalence of comorbidity by specific diseases. Data are presented as median (interquartile range). Mann–Whitney test was used to compare median prevalence of diabetes mellitus in high-income and lower-income economies. COPD: chronic obstructive pulmonary disease.

28 studies reported the presence of overall comorbidity levels within their dataset. The percentage prevalence of pre-existing comorbidity in patients with empyema was high (median 72%, interquartile range (IQR) 58–83%; figure 2a).

Figure 2a shows the median percentage prevalence of reported comorbidity grouped by organ system affected. The percentage prevalence of smokers in patients with empyema had a median (IQR) 41% (30–51%; data reported in 21 studies). The median (IQR) percentage prevalence of alcohol excess in patients with empyema was 15% (8–25%; 30 studies). The median (IQR) percentage prevalence of respiratory comorbidity in patients with empyema was 20% (16–32%; 17 studies). This was similar to the percentage prevalence of cardiac disease (19%, 15–27%; 21 studies) and higher than the percentage prevalence of malignancy (12%, 8–23%; 32 studies) and liver disease (5%, 3–11%; 33 studies).

Figure 2b shows the median percentage prevalence of different comorbidities by specific disease. The median (IQR) percentage prevalence of hypertension in patients with empyema was 23% (17–38%; 21 studies). This was higher than the percentage prevalence of diabetes (17%, 11–27%; 66 studies), stroke (13%, 5–20%; 21 studies), ischaemic heart disease (11%, 5–16%; 12 studies), chronic obstructive pulmonary disease (11%, 6–20%; 40 studies) and chronic kidney disease (7%, 5–13%; 33 studies). The reported presence of immunosuppressive states was relatively low. The median (IQR) percentage prevalence of HIV was 4% (1–9%; 16 studies), steroid use 4% (2–16%; six studies) and recent chemotherapy in 4% (1–15%; three studies).

Where possible, we compared comorbidity prevalence between high-income and lower-income economies. There were no significant differences between studies reporting from high-income economies compared to low-income economies in prevalence of overall pre-existing comorbidity (median 73% *versus* 58%, p=0.623) or diabetes mellitus (median 20% *versus* 14%, p=0.0835). Comparisons for other specific comorbidities were not attempted due to the paucity of data reported from lower-income economies.

### Patients with pleural infection have long hospital stays

Data on outcome of pleural infection was reported in 125 papers (totalling 192 298 patients). Studies reported long inpatient hospital stays (median 19 days, IQR 13–27; reported in 79 studies, totalling 180 931 patients) and median (IQR) mortality in hospital or within 30 days was 4% (1–11%, from 105 studies totalling 179 031 patients).

Prevalence of patients requiring either fibrinolytic treatment (median 31%, IQR 17–52%; 38 studies, 30 071 patients) or surgery (median 20%, IQR 1–32%; 65 studies, 37 330 patients) were also reported.

**FIGURE 3:**
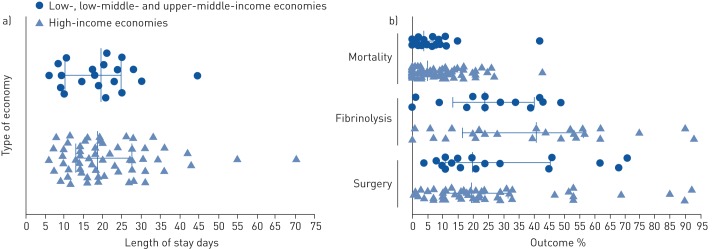
Prevalence of outcomes in studies of patients with pleural infection. Percentage prevalence of outcomes in each study were extracted and data from high-income and lower-income economies were compared. a) Mean length of hospital stay; b) percentage prevalence of mortality, patients requiring fibrinolysis and patients requiring surgical treatment. Data are presented as median (interquartile range).

Figure 3 shows the differences in outcome parameters according to the income category of the country of study. There was no significant difference between studies reporting from high-income compared to lower-income economies in mean length of stay (18.7 days *versus* 19.7 days; figure 3a), percentage prevalence of patients receiving surgery (median 19.5% *versus* 20.0%; figure 3b), fibrinolytic treatment (median 41% *versus* 24%, p=0.1) or 30-day/in-hospital mortality (median 5% *versus* 4%).

## Discussion

This is the first systematic review describing the comorbidities and outcomes of studies reporting on patients with pleural infection since the turn of the 21st century. We found that the percentage prevalence of pre-existing comorbidity was high (median 72%) with a wide range of chronic conditions affecting the major organ systems. This is consistent with large population-based studies, which have reported comorbidity prevalence of up to 74% [5] and supports the hypothesis that the rise in the incidence of pleural infection in recent years might be associated with an increasingly ageing, multi-morbid population.

Chronic respiratory and cardiovascular conditions had the highest percentage prevalence and where specific conditions were reported, hypertension, diabetes mellitus, stroke and ischaemic heart disease all had median prevalence rates between 11% and 23%. As the majority of studies were from high-income settings where these conditions are endemic, this finding may seem unsurprising, and indeed, known risk factors for these diseases including smoking and alcohol excess were also reported (median 42% and 15%, respectively).

When comparing studies from high-income and lower-income economies, there were no significant differences in the prevalence of pre-existing comorbidities. In high-income economies there was a higher prevalence of diabetes mellitus, but this was not significant (median 20% *versus* 14%, p=0.08). The mean age of patients in studies from high-income economies was also significantly higher than from lower-income economies (56.5 *versus* 42.5 years, p<0.001), which probably reflects the longer life expectancy in high-income economies and the increasing prevalence of diabetes mellitus with age [141].

Evidence from large prospective population-based cohort studies will be required to investigate whether there is a causative link between the rise in chronic noncommunicable conditions such as diabetes mellitus and the increased incidence in pleural infection and whether there are true differences between high-income and lower-income economies.

Immunosuppressive states acquired through diseases such as HIV or iatrogenically induced by steroids, immunomodulatory and chemotherapeutic agents can be associated with pleural infection [48, 69]. We found a relatively low prevalence of these conditions among studies of patients with pleural infection; however, this is probably an underestimation as only a small number of studies collected these data. Studies from lower-income economies reported higher levels of HIV compared to high-income economies (median 14% *versus* 4%), in keeping with current HIV trends, but this was not statistically significant [142]. Future studies should focus on the routine collection of these data to better understand the risk, pathogenesis and outcomes of pleural infection in these specific groups where the microbiological milieu and immune response are likely to differ from those in immunocompetent persons.

When analysing outcomes of pleural infection, we confirmed that patients have long hospital stays (median 19 days), comparable to previously reported length-of-stay data [143, 144] This supports the premise that pleural infection is an important use of healthcare resources.

We found a median in-hospital/30-day mortality of 4%, which is lower than has been previously reported. The British Thoracic Society guidelines quote mortality at 20% [145], based on data from a large prospective UK cohort of patients reported in 1996 with pleural infection with an 18% mortality rate at 6 months [143] and the MIST-1 (Multicentre Intrapleural Sepsis Trial) cohort in which 12% had died by 1 year [146]. A recent outcome study of pleural infection of >600 patients from Australia found 1-year mortality of 32.4% in patients with community-acquired pleural infection [147]. We recorded 30-day or in-hospital mortality rather than 3, 6 or 12 months, as this was most commonly reported in the studies we analysed. Our finding of a median 4% in-hospital mortality is consistent with a prospective single centre study of pleural infection reported in 1999, where in-hospital deaths were 4.7%, rising to 14% mortality within 400 days of chest tube insertion [144]. However, in the largest, most recently published population-based cohort study of pleural infection cases in Denmark, overall unadjusted 30-day mortality was reported at between 9% and 10.5% [2]

One explanation for this is that our dataset comprises observational studies with low numbers of participants and significant risk of bias. There was a weakly positive correlation between the percentage mortality reported and number of participants (r=0.2, p=0.05) (supplementary figure S1a). Studies with <300 participants had a lower median mortality than studies with >300 participants (4% *versus* 9%, p=0.072) (supplementary figure S1b) and reported a wider range of mortality estimates. There was no association between numbers of participants and age or year of publication, but as expected there is an association between mortality and age in line with previous data (r=0.35, p=0.0007; data not shown). We were not able to investigate whether there was an association between comorbidity and outcome as the data were not reported in such a way in the original studies.

Studies with more participants are less susceptible to inclusion bias, as data are obtained from databases using International Classification of Diseases (ICD)-10 codes assigned at hospital discharge rather than locally curated cohorts. These larger studies therefore likely provide more reliable mortality estimates than our combined unadjusted estimate, and this is a weakness of our approach. Overall our analysis supports the seriousness of pleural infection, but probably underestimates the true mortality prevalence.

Despite a significant difference in mean age of patients in studies from high- and lower-income economies, the length of hospital stay, percentage of patients requiring surgery and 30-day/in-hospital mortality were similar between groups. Studies from higher-income economies reported a trend towards increased use of fibrinolytics (median 41% *versus* 24%), which offers an option for symptomatic drainage of loculated pleural effusions in patients who are not suitable for surgery. This is consistent with a trend towards the increasing use of fibrinolytics over time in one high-income-based cohort [4]. However, prospective randomised trials have failed to show a mortality benefit from fibrinolytic treatment, and further studies are needed to explore this area of practice [7, 86].

This study describes the results of 134 articles reporting data from >200 000 patients, and thus is the most comprehensive work to date examining the comorbidities and outcomes of patients with pleural infection worldwide. However, the findings may not be generalisable to all settings or fully representative of real-world trends, as the majority of studies are relatively small retrospective observational cohorts from secondary care institutions in high-income settings. Mixed cohorts were included in the analysis to maximise inclusion, and therefore some of the results will reflect patient populations with a proportion of tuberculosis, post-surgical and childhood empyema, which differ in aetiology and outcome to adult bacterial pleural infection.

In conclusion, this study confirms that pleural infection remains an important disease. Patients have a high prevalence of pre-existing comorbidity and are older in high-income economies. Importantly this study highlights the paucity of data on pleural infection from lower-income economies and calls for large prospective registries at the population level in these settings to better understand regional trends in pleural infection and to enable optimal resource provision.

## Supplementary material

10.1183/13993003.00541-2019.Supp1 **Please note:** supplementary material is not edited by the Editorial Office, and is uploaded as it has been supplied by the author.
Supplementary material ERJ-00541-2019.SUPPLEMENT

## Shareable PDF

10.1183/13993003.00541-2019.Shareable1 This one-page PDF can be shared freely online.
Shareable PDF ERJ-00541-2019.Shareable
