# Supplementary material for: A systematic review of comorbidities and outcomes of adult patients with pleural infection
Source: Eur Respir J. 2019 Oct 1;54(3):1900541. doi: 10.1183/13993003.00541-2019 (PMC6860993; doi:10.1183/13993003.00541-2019)
Supplement: Supplementary file 1 [file ERJ-00541-2019.SUPPLEMENT.pdf]

Supplementary table S1: Characteristics of included studies

Study	Region	Economy	Study type	Sample size *	Population studied included cases of TB, paediatric or post-surgical empyema	Comorbidity reported	Outcome reported
Abu-Daff et al., 2013	North America	High income	Retrospective observational cohort	163	N	N	Y
Ahmed et al., 2006	North America	High income	Prospective observational cohort (population based)	24	N	Y	Y
Ahmed et al., 2016	Middle East	Low, low-middle or upper-middle income	Randomised controlled trial	78	N	Y	Y
Akhan et al., 2007	Middle East	Low, low-middle or upper-middle income	Retrospective observational cohort	93	Y	N	Y
Alegre et al., 2002	South Europe	High income	Retrospective observational cohort	36	N	Y	N
Alemán et al., 2015	South Europe	High income	Randomised controlled trial	99	N	Y	Y
Andrade-Alegre et al., 2008)	Central South America	High income	Retrospective observational cohort	33	Y	Y	Y
Anstadt et al., 2003	North America	High income	Retrospective observational cohort	93	N	Y	Y
Asai et al., 2017	East Asia	High income	Retrospective observational cohort	48	N	Y	Y
Baek et al., 2017	East Asia	High income	Retrospective observational cohort	149	Y	Y	Y
Bagheri et al., 2013	Middle East	Low, low-middle or upper-middle income	Randomised controlled trial	40	Y	N	Y
Bagheri et al., 2016	Middle East	Low, low-middle or upper-middle income	Prospective observational cohort	50	Y	N	Y
Barmparas et al., 2009	North America	High income	Retrospective observational cohort (population based)	57	N	N	Y
Banga et al., 2004	India	Low, low-middle or upper-middle income	Retrospective observational cohort	31	Y	Y	Y
Bar et al., 2010	Middle East	High income	Retrospective observational cohort	119	Y	Y	Y
Barthwal et al., 2004	India	Low, low-middle or upper-middle income	Prospective observational cohort	30	Y	N	Y

Bilgin et al., 2006	South Europe	Low, low-middle or upper-middle income	Randomised controlled trial	70	N	N	Y
Birkenkamp et al., 2016	North America	High income	Retrospective observational cohort	91	N	Y	Y
Bongiolatti et al., 2017	South Europe	High income	Retrospective observational cohort	64	Y	Y	Y
Bouros et al., 2002	South Europe	High income	Retrospective observational cohort	20	N	Y	Y
Boyanova et al., 2004	East Europe	Low, low-middle or upper-middle income	Retrospective observational cohort	198	Y	Y	N
Brutsche et al., 2005	West Europe	High income	Retrospective observational cohort	127	Y	N	Y
Casali et al., 2009	South Europe	High income	Retrospective observational cohort	119	Y	N	Y
Caviezel et al., 2017	West Europe	High income	Diagnostic accuracy study	38	N	Y	Y
Chalmers et al., 2009	North Europe	High income	Prospective observational cohort	92	N	Y	Y
Chan et al., 2007	East Asia	High income	Prospective observational cohort	77	Y	Y	Y
Chen et al., 2000	East Asia	High income	Retrospective observational cohort	171	Y	Y	Y
Chen et al., 2007	East Asia	High income	Retrospective observational cohort	102	N	Y	Y
Chen et al., 2009	East Asia	High income	Retrospective observational cohort	141	N	Y	Y
Chen et al., 2014	East Asia	High income	Retrospective observational cohort	602	N	Y	Y
Chen and Hsu, 2013	East Asia	High income	Retrospective observational cohort	141	N	N	Y
Cheng and Vintch, 2005	North America	High income	Retrospective observational cohort	72	N	Y	Y
Chu et al., 2001	North America	High income	Retrospective observational cohort	34	N	N	Y
Davoodabadi et al., 2008	Middle East	Low, low-middle or upper-middle income	Retrospective observational cohort	34	Y	N	Y
de Souza et al., 2000	North America	High income	Retrospective observational cohort	58	Y	Y	Y

Desai and Amadi, 2001	Africa	Low, low-middle or upper-middle income	Retrospective observational cohort	85	Y	Y	Y
Diacon et al., 2004	Africa	Low, low-middle or upper-middle income	Randomised controlled trial	53	Y	Y	Y
El Solh et al., 2007	North America	High income	Retrospective observational cohort	169	N	Y	Y
Eren et al., 2008	Middle East	Low, low-middle or upper-middle income	Retrospective observational cohort	71	Y	N	Y
Farjah et al., 2007	North America	High income	Retrospective observational cohort (population based)	4424	N	N	Y
Finley et al., 2008	North America	High income	Retrospective observational cohort (population based)	11294	Y	N	Y
Froudarakis et al., 2008	South Europe	High income	Prospective observational cohort	20	N	N	Y
Gokce et al., 2009	Middle East	Low, low-middle or upper-middle income	Retrospective observational cohort	93	Y	N	Y
Grijalva et al., 2011	North America	High income	Retrospective observational cohort (population based)	130969	N	N	Y
Hagos et al., 2016	Africa	Low, low-middle or upper-middle income	Retrospective observational cohort	35	Y	N	Y
Hajjar et al., 2016	Middle East	High income	Retrospective observational cohort	63	N	Y	Y
Hardavella et al., 2017	South Europe	High income	Retrospective observational cohort	84	N	Y	Y
Heimes et al., 2017	North America	High income	Retrospective observational cohort	103	N	Y	Y
Herrera-Kiengelher et al., 2010	Central South America	Low, low-middle or upper-middle income	Retrospective observational cohort	347	N	Y	Y
Hooper et al., 2015	North Europe	High income	Randomised controlled trial	35	N	N	Y
Hoth et al., 2003	North America	High income	Retrospective observational cohort	37	Y	N	Y
Hsiao et al., 2017	East Asia	High income	Retrospective observational cohort	33	N	Y	Y
Hsieh et al., 2008	East Asia	High income	Retrospective observational cohort	71	Y	Y	Y

Huang et al., 2010	East Asia	Low, low-middle or upper-middle income	Retrospective observational cohort	259	Y	Y	Y
Jiménez et al., 2006	South Europe	High income	Prospective observational cohort	50	N	N	Y
Kearney et al., 2000	North Europe	High income	Retrospective observational cohort	50	N	N	Y
Keeling et al., 2008	North Europe	High income	Retrospective observational cohort	52	N	N	Y
Khwaja et al., 2005	North America	High income	Retrospective observational cohort	21	N	Y	Y
Kim et al., 2004	East Asia	High income	Retrospective observational cohort	70	Y	N	Y
Kim et al., 2014	East Asia	High income	Retrospective observational cohort	127	N	Y	N
Kim et al., 2016	East Asia	High income	Retrospective observational cohort	158	N	Y	Y
Koma et al., 2017	East Asia	High income	Retrospective observational cohort	81	N	Y	Y
Kundu et al., 2010	India	Low, low-middle or upper-middle income	Prospective observational cohort	46	Y	Y	Y
Lardinois et al., 2005	West Europe	High income	Retrospective observational cohort	328	Y	N	Y
Lee et al., 2015	East Asia	High income	Prospective observational cohort	32	N	Y	Y
Levinson and Pennington, 2007	North America	High income	Retrospective observational cohort	30	N	Y	Y
Li et al., 2017	East Asia	Low, low-middle or upper-middle income	Retrospective observational cohort	209	N	N	Y
Lin et al., 2007	East Asia	High income	Retrospective observational cohort	207	Y	Y	Y
Lin et al., 2009	East Asia	High income	Diagnostic accuracy study	45	N	Y	Y
Luh et al., 2005	East Asia	High income	Retrospective observational cohort	234	Y	Y	Y
Majid et al., 2016	North America	High income	Retrospective observational cohort	73	N	Y	Y
Malhotra et al., 2007	India	Low, low-middle or upper-middle income	Prospective observational cohort	76	Y	Y	Y

Petrusevska-Marinkovic et al., 2016	East Europe	Low, low-middle or upper-middle income	Retrospective observational cohort	44	N	Y	Y
Marks et al., 2012	North Europe	High income	Retrospective observational cohort	406	Y	Y	Y
Marra et al., 2012	West Europe	High income	Retrospective observational cohort	61	Y	Y	Y
Maskell et al., 2005	North Europe	High income	Retrospective observational cohort	430	Y	Y	Y
McClune et al., 2016	North America	High income	Retrospective observational cohort	101	Y	N	Y
Medeiros et al., 2012	Central South America	Low, low-middle or upper-middle income	Retrospective observational cohort	52	Y	Y	Y but excluded due to overlapping dataset
Mehta et al., 2016	North America	High income	Prospective observational cohort	55	N	Y	Y
Melloni et al., 2004	South Europe	High income	Retrospective observational cohort	40	N	Y	Y
Metaxas et al., 2007	South Europe	High income	Retrospective observational cohort	106	Y	N	Y
Metin et al., 2010	Middle East	Low, low-middle or upper-middle income	Retrospective observational cohort	114	Y	Y	Y
Mikkola et al., 2010	North Europe	High income	Retrospective observational cohort	143	N	Y	Y
Misthos et al., 2005	South Europe	High income	Randomised controlled trial	127	N	N	Y
Monteiro et al., 2011	South Europe	High income	Retrospective observational cohort	27	N	Y	Y
Muhammad, 2012	Africa	Low, Low-middle or Upper-middle income	Prospective observational cohort	69	N	N	Y
Nadir et al., 2007	Middle East	Low, low-middle or upper-middle income	Retrospective observational cohort	68	Y	N	Y
Nakamoto et al., 2016	East Asia	High income	Prospective observational cohort	20	Y	Y	Y

Nakamura et al., 2010	East Asia	High income	Retrospective observational cohort	31	N	Y	Y
Nandeesh et al., 2013	India	Low, Low-middle or Upper-middle income	Prospective observational cohort	40	Y	N	Y
Nielsen et al., 2011	North Europe	High income	Retrospective observational cohort	158	Y	Y	Y
O'Connor et al., 2013	North America	High income	Retrospective observational cohort	125	N	N	Y
Ohuchi et al., 2014	East Asia	High income	Retrospective observational cohort	29	N	Y	Y
Okada et al., 2000	East Asia	High income	Randomised controlled trial	24	Y	N	Y
Okiror et al., 2014	North Europe	High income	Retrospective observational cohort	107	N	Y	N
Ozol et al., 2006	Middle East	Low, low-middle or upper-middle income	Retrospective observational cohort	107	N	Y	Y
Park et al., 2016	East Asia	High income	Retrospective observational cohort	421	N	Y	N
Petrakis et al., 2004	South Europe	High income	Retrospective observational cohort	38	N	N	Y
Pilav et al., 2009	East Europe	Low, low-middle or upper-middle income	Retrospective observational cohort	100	N	N	Y
Podbielski et al., 2000	North America	High income	Retrospective observational cohort	30	N	N	Y
Popowicz et al., 2017	Australasia	High income	Prospective observational cohort	61	N	Y	Y
Porcel et al., Lung 2017	South Europe	High income	Retrospective observational cohort	62	N	Y	Y but excluded as overlapping dataset
Porcel et al.,Respiraology 2017	South Europe	High income	Retrospective observational cohort	90	N	N	Y
Potaris et al., 2007	South Europe	High income	Retrospective observational cohort	122	Y	Y	Y
Powell et al., 2000	North America	High income	Retrospective observational cohort	70	N	Y	Y

Rahman et al., 2011	North Europe	High income	Randomised controlled trial	210	N	Y	Y
Ravaglia et al., 2012	South Europe	High income	Retrospective observational cohort	41	Y	N	Y
Roberts, 2003	North America	High income	Retrospective observational cohort	172	Y	N	Y
Schweigert et al., 2016	West Europe	High income	Retrospective observational cohort	335	N	Y	Y
Shankar et al., 2000	India	Low, low-middle or upper-middle income	Retrospective observational cohort	103	Y	Y	Y
Shen et al., 2012	East Asia	High income	Retrospective observational cohort	26385	N	Y but excluded due to overlapping dataset	Y included
Shin et al., 2013	East Asia	High income	Retrospective observational cohort	111	Y	Y	Y
Smolikov et al., 2006	Middle East	High income	Retrospective observational cohort	71	N	N	Y
Solaini et al., 2007	South Europe	High income	Retrospective observational cohort	120	Y	Y	Y
Søgaard et al., 2014	North Europe	High income	Retrospective observational cohort (population based)	6878	Y	Y	N
Soriano et al., 2005	South Europe	High income	Prospective observational cohort	112	N	Y	Y
Rodríguez Suárez et al., 2012	South Europe	High income	Retrospective observational cohort	210	N	Y	Y
Swart, 2002	Africa	Low, low-middle or upper-middle income	Retrospective observational cohort	77	Y	Y	Y
Tantraworasin et al., 2018	East Asia	Low, low-middle or upper-middle income	Retrospective observational cohort	328	N	Y	N
Terra et al., 2012	Central South America	Low, low-middle or upper-middle income	Retrospective observational cohort	206	Y	N	Y
Thommi et al., 2007	North America	High income	Retrospective observational cohort	52	N	N	Y
Thommi et al., 2012	North America	High income	Randomised controlled trial	68	N	Y	Y

Tsai et al., 2016	East Asia	High income	Retrospective observational cohort	33	Y	N	Y
Tsang et al., 2007	East Asia	High income	Retrospective observational cohort	63	N	Y	Y
Tsujimoto et al., 2015	East Asia	High income	Retrospective observational cohort	36	N	Y	N
Tuncozgur et al., 2001	Middle East	Low, low-middle or upper-middle income	Randomised controlled trial	49	N	N	Y
Vaziri and Abed, 2012	Middle East	Low, low-middle or upper-middle income	Retrospective observational cohort	112	Y	Y	Y
White et al., 2013	North America	High income	Retrospective observational cohort	187	Y	Y	Y
Wong and Yap, 2016	Australasia	High income	Retrospective observational cohort	108	N	Y	Y
Wozniak et al., 2009	North America	High income	Retrospective observational cohort	104	N	Y	Y
Wu et al., 2015	East Asia	High income	Retrospective observational cohort (population based)	34250	N	Y	N
Wurnig et al., 2006	West Europe	High income	Retrospective observational cohort	130	Y	N	Y
Yamaguchi et al., 2009	East Asia	High income	Retrospective observational cohort	26	Y	Y	Y
Pefura Yone et al., 2012	Africa	Low, low-middle or upper-middle income	Prospective observational cohort	55	N	Y	Y

* Where the same study reported both comorbidity and outcome data, if sample sizes differed the largest samples size is recorded.

Supplementary table S2: comorbidities

Comorbidity	Number of patients included in the analysis reported in studies from high income economies	Number of patients reported in studies from lower income economies
Any comorbidity	9417	488
Cardiac	2269	0
Respiratory	2495	326
Malignancy	3393	576
Liver	4357	503
Current smoker	2009	406
Alcohol excess	3462	743
Chemotherapy	735	0
Steroid therapy	828	46
Asthma	617	642
Chronic obstructive pulmonary disease	37629	928
Congestive cardiac failure	35411	320
Ischaemic heart disease	35716	78
Hypertension	36172	639
Diabetes mellitus	40711	1693
Human Immunodeficiency virus	1709	720
Chronic Kidney Disease	4142	542
Stroke	36076	785

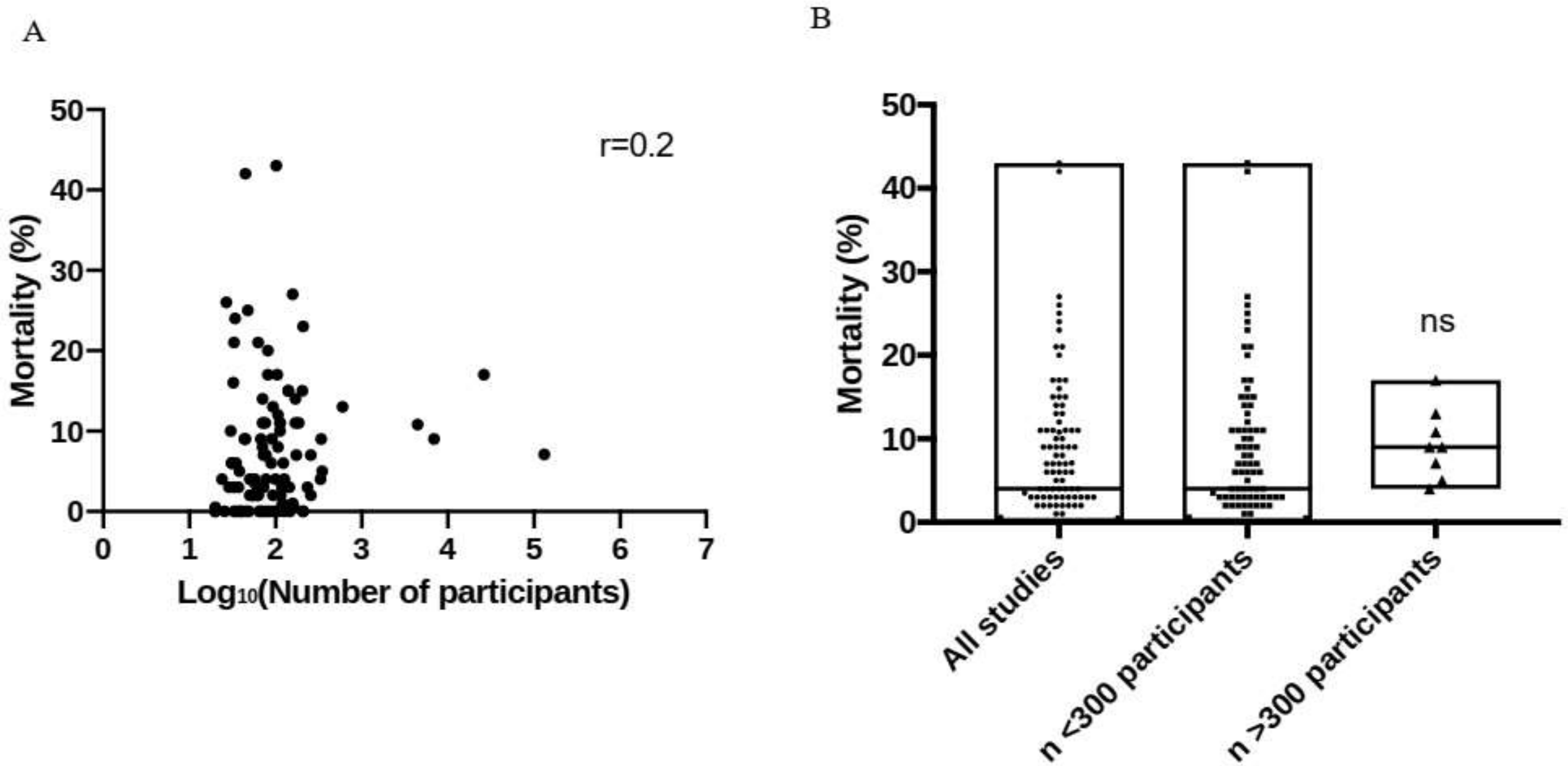

Supplementary Figure S1: Percentage prevalence of in-hospital/30-day mortality of patients with pleural infection. A: Correlation plot showing log transformed number of participants plotted against percentage mortality. Spearman rank r value shown, *= p<0.05. **B:** Scatter plot showing percentage mortality for all included studies (left bar), studies with under 300 participants (middle bar) and over 300 participants (right bar). Median and range shown. Mann-Whitney test to compare median mortality of all included data sets with dataset with over 300 participants, ns = not significant
