# Supplementary material for: A systematic review of comorbidities and outcomes of adult patients with pleural infection
Source: Eur Respir J. 2019 Oct 1;54(3):1900541. doi: 10.1183/13993003.00541-2019 (PMC6860993; doi:10.1183/13993003.00541-2019)
Supplement: Supplementary file 2 [file ERJ-00541-2019.Shareable.pdf]

SHAREABLE PDF

A systematic review of comorbidities and outcomes of adult patients with pleural infection

Tamsin N. Cargill[1,4], Maged Hassan[1,4], John P. Corcoran[2], Elinor Harriss[3], Rachelle Asciak[1], Rachel M. Mercer[1], David J. McCracken[1], Eihab O. Bedawi[1] and Najib M. Rahman[1]

Affiliations: [1]Oxford Centre for Respiratory Medicine, Oxford University Hospitals NHS Trust, Oxford, UK. [2]Interventional Pulmonology Service, Respiratory Medicine Dept, University Hospitals Plymouth, Plymouth, UK. [3]Bodleian Health Care Libraries, University of Oxford, Oxford, UK. [4]Joint first authors.

Correspondence: Maged Hassan, Oxford Centre for Respiratory Medicine, Churchill Hospital, Old Road, Oxford, OX3 7LE, UK. E-mail: magedhmf@gmail.com

@ERSpublications
In pleural infection, patients from higher-income countries tend to be older with more comorbidities and are more likely to be referred for fibrinolytic treatment in comparison to patients from lower-income countries http://bit.ly/2K2M5HL

Cite this article as: Cargill TN, Hassan M, Corcoran JP, *et al.* A systematic review of comorbidities and outcomes of adult patients with pleural infection. *Eur Respir J* 2019; 54: 1900541 [https://doi.org/10.1183/13993003.00541-2019].

This single-page version can be shared freely online.

ABSTRACT
Background: Pleural infection remains an important cause of mortality. This study aimed to investigate worldwide patterns of pre-existing comorbidities and clinical outcomes of patients with pleural infection.
Methods: Studies reporting on adults with pleural infection between 2000 and 2017 were identified from a search of Embase and MEDLINE. Articles reporting exclusively on tuberculous, fungal or post-pneumonectomy infection were excluded. Two reviewers assessed 20 980 records for eligibility.
Results: 211 studies met the inclusion criteria. 134 articles (227 898 patients, mean age 52.8 years) reported comorbidity and/or outcome data. The majority of studies were retrospective observational cohorts (n=104, 78%) and the most common region of reporting was East Asia (n=33, 24%) followed by North America (n=27, 20%). 85 articles (50 756 patients) reported comorbidity. The median (interquartile range (IQR)) percentage prevalence of any comorbidity was 72% (58–83%), with respiratory illness (20%, 16–32%) and cardiac illness (19%, 15–27%) most commonly reported. 125 papers (192 298 patients) reported outcome data. The median (IQR) length of stay was 19 days (13–27 days) and median in-hospital or 30-day mortality was 4% (IQR 1–11%). In regions with high-income economies (n=100, 74%) patients were older (mean 56.5 *versus* 42.5 years, p<0.0001), but there were no significant differences in prevalence of pre-existing comorbidity nor in length of hospital stay or mortality.
Conclusion: Patients with pleural infection have high levels of comorbidity and long hospital stays. Most reported data are from high-income economy settings. Data from lower-income regions is needed to better understand regional trends and enable optimal resource provision going forward.

Copyright ©ERS 2019. This article is open access and distributed under the terms of the Creative Commons Attribution Licence 4.0.
